# Study on the Heat Reduction Effect of Biomimetic Unidirectional Transporting Channels Inspired by *Nepenthes alata*

**DOI:** 10.3390/biomimetics4040070

**Published:** 2019-10-15

**Authors:** Yixuan Zhang, Deyuan Zhang, Dongyue Wang, Xiangyu Zhang

**Affiliations:** 1 School of Mechanical Engineering and Automation, Beihang University, Beijing 100091, China; zhangyixuan@buaa.edu.cn (Y.Z.); zhangdy@buaa.edu.cn (D.Z.); wangdy@buaa.edu.cn (D.W.); 2 Institute of Bionic and Micro-Nano Systems, Beihang University, Beijing 100091, China; 3 Beijing Advanced Innovation Center for Biomedical Engineering, Beihang University, Beijing 100091, China

**Keywords:** heat management, cooling, micro channels, directional water transport

## Abstract

Heat control has been a momentous problem in engineering areas which include manufacturing, aeronautics, microchips and so forth for a considerable amount of time. The control of material for thermal deformation, effective cooling are the key components of the aero crafts and compactly laid out microchips are urgently needed for improvement. In a micro-scale, researchers are mainly focused on the mechanism, design, improvement and heat transfer of straight channels other than developing other types of channels. A previous study on the carnivorous plant, *Nepenthes alata*, indicates that the water can be transported continuously and directionally on the surface of the rim of the pitcher because of its multi-scale structures. Meanwhile, the transporting speed is much higher than what was thought previously. Inspired by this unique phenomenon, the heat management ability of this biological micro channel is investigated in this research. Firstly, based on existing studies, the features of the biological channels are extracted. Then, the unidirectional channels are designed and fabricated by elliptical vibration cutting accordingly. The experimental platform for thermal control was established consequently. Both bio-inspired and straight triangular channels of the same depth and width were set for comparison. Through the comparative experiments, it is concluded preliminarily that the critical point of heat transfer performance of the two channels exists, and the biomimetic structure can improve and strengthen the cooling effects at a large flow rate because of the unique geometric structure. The temperature reduction of the bio-inspired channels can be increased by up to 84 percent compared with straight channels in a single experiment when heated up to 150 centigrade.

## 1. Introduction

Heat control is significant in engineering areas, especially for microchips and MEMS system. As the technology advances, the systems are designed more compactly and more heat is generated, lowering the control stability. However, heat causes temperature noise which influences the accuracy of the measurement system. Therefore, micro cooling devices are in demand to avoid rapid temperature rises and uneven temperature distribution [1].

Micro scale heat transfer has been an extensively discussed topic during the last 20 years [2]. The mechanism, design, improvement and heat transfer of parallel straight micro channels have been numerically and experimentally studied extensively [3,4]. Kim et al [5] studied the annular condensation heat transfer over micro channels with a hydraulic diameter of 1 mm and announced that maintaining annular flow in a large fraction of the micro channels with thin liquid film is the crux to high condensation heat transfer. Naphon et al discovered the geometry configuration and the size of roughness irregularities of the micro channels is significant to the enhancement of heat transfer [6], and Sahar et al found aspect ratio is not a major factor by numerically analyzing the single-phase flow and heat transfer in single channel [7]. While the heat transfer performance of deep channels is proved to be better than that of shallow channels theoretically and experimentally [8,9,10].

Researchers come up with other ideas to enhance the heat transfer performance in a micro scale instead of adjusting geometry parameters of the straight channels. Suspended nano particles are added to the liquid to increase thermal properties as the thermal conductivity of solids is better than that of liquid. There is a significant rise in the heat transfer performance using nanofluid as the transfer media instead of deionized water at the same Reynolds number [11,12]. There are differences between the various types, shapes and volume fractions of particles [12,13]. Thermal conductivity of the suspended nanofluid can increase by over 20% with low particle concentrations (1–5 vol%) [14,15,16].

Other studies indicate that the alteration of the shape of the channels can enhance the heat transfer performance. The micro-heat-exchanger using porous channels are shown to be better in heat transfer than using micro channels [17]. Huang et al studied the three types of micro channel heat sinks, finding the setup of the slot structure in the micro-channel heat sinks can improve the heat transfer and the performance of trapezoidal stagger-slot channels was the best [18]. And the design of ladder shaped micro channels by Brinda et al [19] decreases the thermal resistance and increases heat transfer coefficient. However, the channels mentioned above are mainly fabricated by lithography, wire cutting and laser beam machining. These manufacturing methods are very effective and economic while processing simple structures in micro scale. But when it comes to repeated complex structures, this technology becomes inefficient and costly. Therefore, the alternation is restricted. As the number of studies on different types of channels is still limited, the design of the different types of micro channels is still noteworthy. This research discusses the heat transfer performance of micro-channels inspired by *Nepenthes alata*.

**Figure 1:**
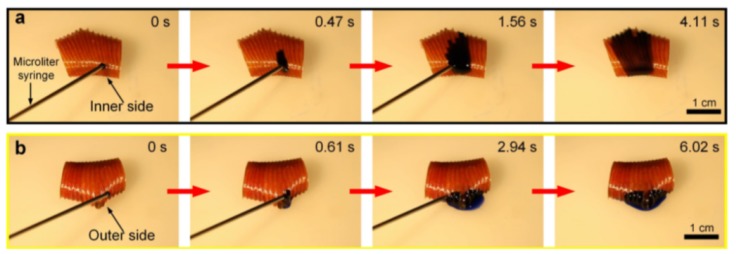
Directional water transport on nature peristome surface of *Nepenthes alata* [20].

Directional water transport was discovered on the rim of the pitcher of the carnivorous plant, *Nepenthes alata* [20]. The multiscale structure which optimizes and enhances the capillary rise in the transport direction and pins the back flow on the surface plays a leading role in unidirectional water transport. However, the transporting speed is much higher than what was thought previously and the anti-gravity water transport can be realized without external added energy (Figure 1).

Inspired by this unique phenomenon, a bio-inspired micro channels was designed and the heat management ability of this biological micro channel was investigated in this research. By comparing the heat transfer performance of the bio-inspired channels and the straight channels in the experiments, the biomimetic channels have proven to hold better temperature reduction performance in certain conditions. This provides a foundation to design a micro-heat-exchanger without external power working on an irregular surface.

## 2. Materials and Methods

### 2.1. Materials and Elliptical Vibration Machining (EVM)

The experiments take poly tetra fluoroethylene (PTFE), as a heat transfer medium whose thermal conductivity is 0.24, respectively. The bio-inspired micro channels (BIMC) on PTFE and PMMA were fabricated by the EVM extrusion method [21] and PDMS test samples were made by replicating channels on PTFE.

**Figure 2:**
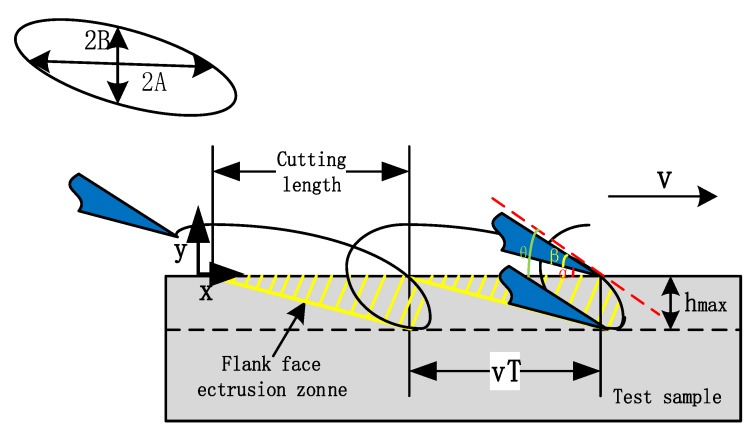
The trajectory of extrusion elliptical vibration machining (EVM) cutting.

The cutting tool motion trajectory (Figure 2) can be defined as follows:

(1) $$\left\{ \begin{array}{l} x=A\sin(2\pi ft+\phi )+vt\\ y=B\sin(2\pi ft) \end{array}\right.$$

where *x* and *y* are the tool displacement along the cutting direction (μm) and the tool displacement along the cutting depth direction (μm), respectively, *A* and *B* are the toll vibration amplitude in the cutting direction and the cutting depth direction, respectively and *f*, *ϕ*, *v* and *t* are the tool vibration frequency (Hz), the phase shift between the vibration of the *x*-axial and *y*-axial directions (rad), the cutting velocities (μm/s) and the time (s), respectively.

In this experiment, the cutting length between the adjacent tool trajectories L was 450 μm, and the maximum cutting depth was 120 μm. The frequency of the vibration was set as 3/4 Hz and the cutting velocity was 450 μm/s. The phase shift between the *x* and *y* directions was set. The final trajectory is as follows:

(2) $$\left\{ \begin{array}{l} x=450\sin(\frac{3}{2}\pi t+\frac{1}{6}\pi )+450t\\ y=-120\sin(\frac{3}{2}\pi t) \end{array}\right.$$

### 2.2. Experimental Design and Set Up

**Figure 3:**
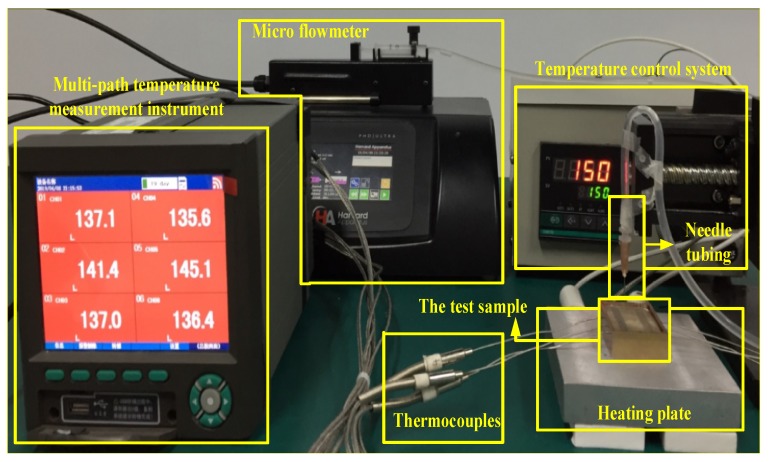
Experiment platform.

The experiment platform is shown as Figure 3. Absolute ethanol was used as the heat transfer media.

All the test samples for experiments had the same geometry parameters of 40 × 30 × 10 mm. Each test sample had five parallel bio-inspired micro channels (BIMC) and the control group had five parallel straight triangular micro channels (STMC) that were set symmetrically (Figure 3). The holes whose diameter was 1.2 mm are drilled every 5 mm on both the lateral sides of the sample. The holes were 7.5 mm in depth so that the inserted thermocouples can test the temperature of the precise middle of the channels. The axis of the holes were 1.7 mm to the surface of the sample (0.6 mm for the radius of the drilled holes, 0.2 mm for structures in the channels and 0.9 mm for interval) to avoid the tilt of drilling damaging the structures in the channels and to keep the distance to the surface as short as possible in order to get the undistorted temperature data of the channels.

**Figure 4:**
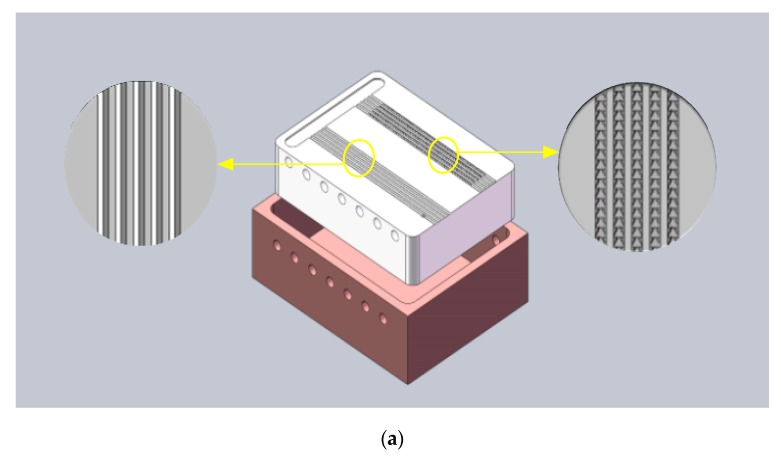

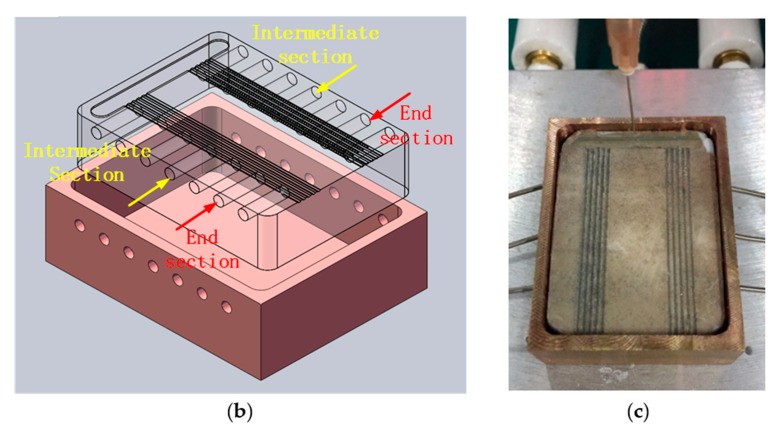
(**a**) The model of the test sample and the container; (**b**) The model shows the tested holes of the intermediate and end section. (**c**) The picture of the test sample and the container with thermocouples.

A cooper container of 2 mm thick was designed for the experiment to reduce heat loss (Figure 4a). The size of the inner surface of the container was fully fit with the sample. The holes of 2 mm in diameter were drilled for the insertion of the thermocouples. A hole of 5 mm in diameter was drilled on the bottom, and therefore the sample could be withdrawn conveniently after the experiment.

A heating plate made of cast aluminum, which can be heated up to 300 centigrade, was placed beneath the container for heating. A thermocouple was connected at the rear to monitor the temperature. The temperature control system was connected to the thermal plate. The resolution of the temperature control system is 1 centigrade.

K type thermocouples were used in the experiment. The nominal diameter of the thermocouple was 1 mm. During the experiment, the thermocouples were inserted in the holes drilled on the lateral side of the sample. As the thermal resistance of gas is ordinarily higher than solid, the end of the thermocouples should touch the bottom of the drilled holes in order to test the precise temperature of the channels. A multi-path temperature measurement instrument was connected to the thermocouples. The temperature data updated every 4 seconds and 6-path real time temperature can be shown on the screen. The historical data can be converted into excel for data processing.

The micro flowmeter can provide a liquid flow ranging from 7.23324 nL/min to 3.75573 mL/min, using syringes with a diameter ranging from 1 to 5 mm. A syringe with a capacity of 2.5 mL was mounted on the micro flowmeter for liquid injection. The hose joint to the syringe channels, and the liquid to needle tubing hung above the test sample. The flux of the absolute ethanol varied from 50 μL/min to 1000 μL/min.

## 3. Results

### 3.1. Temperature Variation of the Test Sample

In this experiment, absolute ethanol was used as a medium. Its tested evaporation exists in both BIMC and STMC with a heating temperature above 90 °C. The temperatures of the entrance, intermediate section and the end section of the channels were tested. However, the temperature change of the entrance was unstable due to the complex flow condition of the section. Therefore, the temperature variation of this section has not being discussed in this paper.

**Figure 5:**
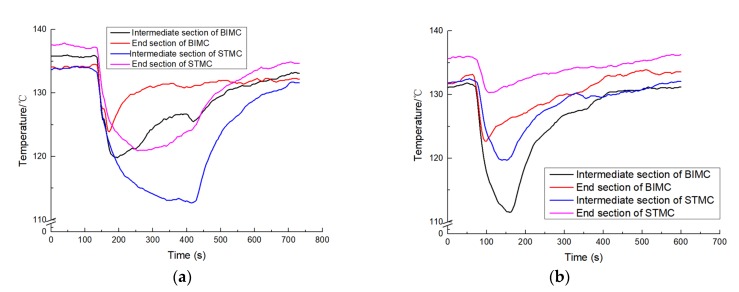
(**a**) The temperature variation with liquid flow of 200 μL/min; (**b**) The temperature variation with liquid flow of 600 μL/min.

The temperature variation of the intermediate section and the end section of BIMC and STMC when heated at 150 °C at the flow rate of 200 μL/min and 600 μL/min are shown in Figure 5 respectively.

In Figure 5a, absolute ethanol is added in the channels when the temperature stabilizes at 132 to 138 °C. The temperatures of both channels dropped rapidly as the liquid was added. The liquid stopped when the temperature variation was no more than 0.3 °C within 30 seconds. The scale of temperature reduction of STMC is considerably larger than that of BIMC with the flow rate of 200 μL/min. In Figure 5b, when the adding flow rate is 600 μL/min, the relative temperature reduction trends changes. The scale of temperature reduction of BIMC becomes larger than that of STMC at both intermediate and end sections.

### 3.2. Temperature Reduction Variation along with the Changing Flow Rate

Further experiments to test the influence of the flow rate to the effects of temperature reduction were then carried out as shown in Table 1. No obvious temperature reduction occurs with the flow rate lower than 200 μL/min and liquid overflows the channels and covers the surface of the test sample when the flow rate is larger than 1000 μL/min. Therefore, only experiment data with the flow rate between 200 μL/min and 1000 μL/min are recorded.

**Figure 6:**
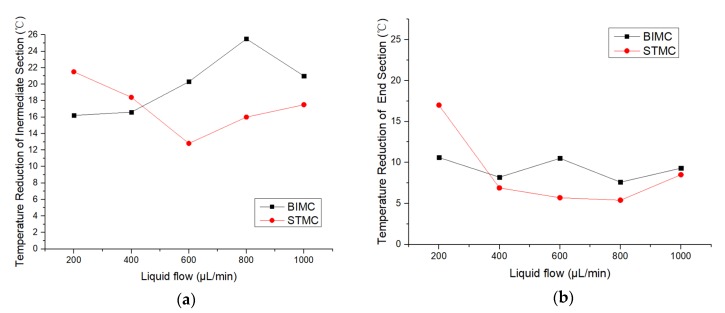
(**a**) The temperature reduction along with the changing flow rate at the intermediate section; (**b**) The temperature reduction along with the changing flow rate at the end section.

$T_{\max}$ is measured when relatively stable without liquid. $T_{\min}$ is measured after stabilizing with liquid added. ΔT indicates the temperature reduction of different sections. No regular pattern is found along with the change of the flow rate. However, the temperature reduction of STMC is larger than that of BIMC at a lower flow rate and lower than the temperature reduction of BIMC at a larger flow rate. The critical points when the temperature reduction of BIMC exceeds STMC occur at both intermediate and end sections of the channels as shown in Figure 6. The critical point indicates the shift of the optimal heat transfer performance from STMC to BIMC.

The experiments were also conducted at the heating temperature of 90 °C and 120 °C. The range of flow rates were selected according to the same principal as the experiment conducted at 150 °C. The evaporation of ethanol is less violent at 90 °C and 120 °C, resulting in a lower range of the flow rate. The temperature reduction is shown in Table 2 and Table 3.

**Figure 7:**
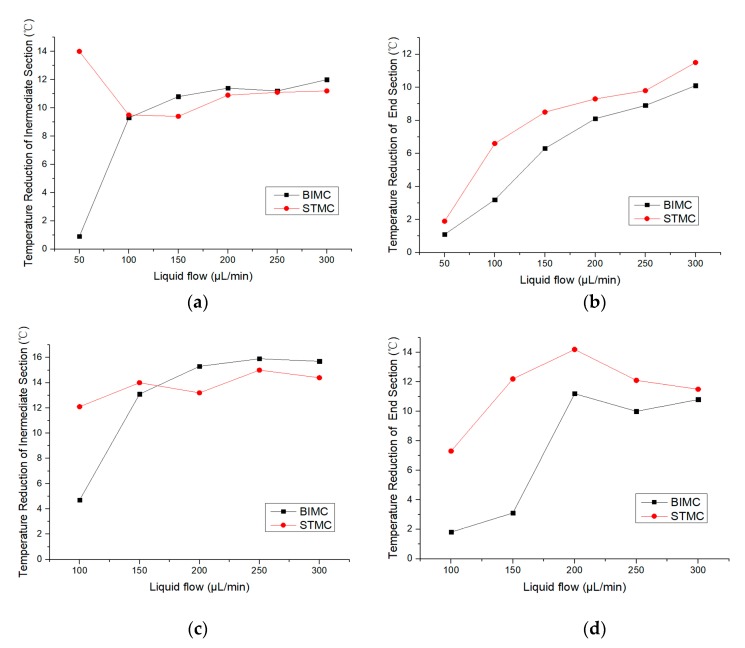
(**a**) Temperature reduction at the intermediate section at the heating temperature of 90 °C; (**b**) Temperature reduction at the end section at the heating temperature of 90 °C; (**c**) Temperature reduction at the intermediate section at the heating temperature of 120 °C; (**d**) Temperature reduction at the end section of the heating temperature of 120 °C.

Figure 7 illustrates the trends of temperature reduction changing with the flow rate of BIMC and STMC respectively. The critical points also occur at the intermediate section when heated up to 90 and 120°C. While the effect of the temperature reduction of STMC maintains better than BIMC at the end section. However, the difference between the effects of two channels declines in general as the flow rate increases. It can be seen that the end section of BIMC and STMC have similar heat transfer at a large flow rate.

The relative coefficient (RC) is defined to indicate the heat transfer performance of BIMC compared with STMC.

$$RC=\frac{\mathrm{the}\;\mathrm{temperaure}\;\mathrm{reduction}\;\mathrm{of}\;\mathrm{BIMC}\;-\;\mathrm{the}\;\mathrm{temperaure}\;\mathrm{reduction}\;\mathrm{of}\;\mathrm{STMC}}{\mathrm{the}\;\mathrm{temperaure}\;\mathrm{reduction}\;\mathrm{of}\;\mathrm{STMC}}\times 100\%$$

**Figure 8:**
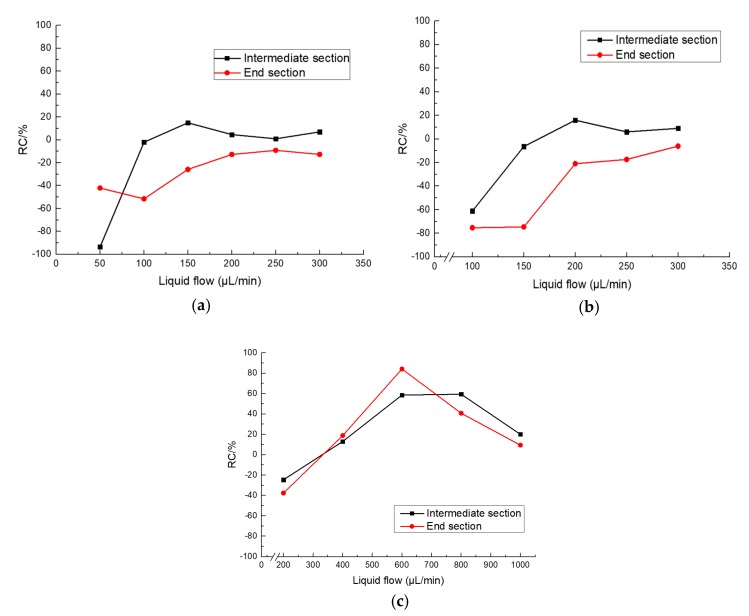
(**a**) T RC at the heating temperature of 90 °C; (**b**) RC at the heating temperature of 120 °C; (**c**) RC at the heating temperature of 150 °C.

The RC changing with the flow rate at different temperatures is shown in Figure 8. The RC over 0 means the heat transfer performance of BIMC is better, while RC below 0 means STMC is better. The flow rate whose RC equals to 0 is the critical point of heat transfer as mentioned above.

In Figure 8a,b, the RC of both channels increases at lower flow rate in general and enters a relatively stable stage with low fluctuation. The critical points only occur at the intermediate section at 90 and 120°C. In Figure 8c, RC also increase sharply at a lower flow rate, but the fluctuation is considerably larger at a larger flow rate. The critical points appear at both the intermediate and end section and the heat transfer performance of BIMC can be 58% and 84% better than STMC at the intermediate and end section respectively.

The flow resistance of BIMC is larger than that of STMC because of the repeated wedge-shaped structure. At low temperatures, the liquid is mainly restricted in the entrance. The mass flow of the liquid evaporates before getting to the intermediate and end section and those sections are only covered with a thin film of ethanol. The liquid flows smoothly in STMC. Therefore, the flow reaching the intermediate and end section of the channel is responsible for the poor temperature reduction performance of BIMC. When the flow rate increases, the unidirectional transports of BIMC take effect and the flowing speed becomes quicker. Besides, the repeated wedge-shaped structures disturb the smooth flowing conditions and enlarge the areas of heat transfer. Those combined reasons cause better heat transfer performance of BIMC and the critical points occur in between them.

## 4. Conclusions

The heat transfer experiment of the biomimetic micro channels was investigated in this paper. The heating temperature ranges from 90 to 150°C and the flow rate varies from 50 to 1000 μL/min during the experiments. Preliminary conclusions can be reached as follows:

1. With the increasing flow rate, the relative coefficient of BIMC compared with STMC also increases in general. The temperature reduction of STMC is better than that of BIMC at a lower flow rate and the critical points when the heat transfer performance of BIMC exceeds STMC.
2. The existence of a critical point is associated with both the heating temperature and transporting distance of the channels.
3. The high heating temperature can have better heat transfer performance for BIMC. The temperature reduction can be increased by 84 percent with the flow rate of 600 μL/min at the heating temperature of 150 °C.

In conclusion, compared to STMC, the appropriate application range for BIMC is 400 to 1000 μL/min at the heating temperature of 150 °C, 100 to 300 μL/min at 90 °C and 150 to 200 μL/min at 120 °C. Furthermore, this paper provides researchers with a reference for further application of this BIMC.

## Figures and Tables

**Table 1:** Temperature reduction at heating temperature of 150 °C.

Measuring Sections	Temperature Measurement Items/°C	Liquid Flow/μL/min				
		200	400	600	800	1000
Intermediate section of BIMC	$T_{\max}$	136.4	136.3	131.8	133.8	132.8
	$T_{\min}$	116.5	115.5	111.5	108.3	111.8
	ΔT	19.9	20.8	20.3	25.5	21
End section of BIMC	$T_{\max}$	135	134.3	133.2	134.3	133.6
	$T_{\min}$	122.6	109	122.7	126.7	124.3
	ΔT	12.4	25.3	10.5	7.6	9.3
Intermediate 0 of STMC	$T_{\max}$	135	134.9	132.5	131.3	134.9
	$T_{\min}$	113.3	116.5	119.7	115.3	117.4
	ΔT	21.7	18.4	12.8	16	17.5
End section of STMC	$T_{\max}$	137.8	137.4	136	134.9	135.4
	$T_{\min}$	118.5	128.7	130.3	129.5	126.9
	ΔT	19.3	8.7	5.7	5.4	8.5

$T_{\max}$ means the maximum equilibrium temperature; $T_{\min}$ means the minimum equilibrium temperature when cooling; ΔT means the difference between $T_{\max}$ and $T_{\min}$.

**Table 2:** Temperature reduction at heating temperature of 90 °C.

Measuring Sections	Temperature Measurement Items/°C	Liquid Flow/μL/min					
		50	100	150	200	250	300
Intermediate section of BIMC	$T_{\max}$	79.4	79.6	78.4	78.3	78.3	79.4
	$T_{\min}$	78.5	70.3	67.6	66.9	67.1	67.1
	ΔT	0.9	9.3	10.8	11.4	11.2	12.3
End section of BIMC	$T_{\max}$	79.4	79.2	78.9	78.6	78.7	79.3
	$T_{\min}$	78.3	76	72.6	70.5	69.8	69.2
	ΔT	1.1	3.2	6.3	8.1	8.9	10.1
Intermediate section of STMC	$T_{\max}$	80.1	79.9	78.5	78.5	78.5	79.3
	$T_{\min}$	66.1	70.4	69.1	67.6	67.4	67.8
	ΔT	14	9.5	9.4	10.9	11.1	11.5
End section of STMC	$T_{\max}$	78.3	78.3	76.8	76.6	76.9	77.3
	$T_{\min}$	76.4	71.7	68.3	67.3	67.1	65.8
	ΔT	1.9	6.6	8.5	9.3	9.8	11.5

**Table 3:** Temperature reduction at heating temperature of 120 °C.

Measuring Sections	Temperature Measurement Items/°C	Liquid Flow/μL/min				
		100	150	200	250	300
Intermediate section of BIMC	$T_{\max}$	104.5	104.3	104.5	104.4	104.6
	$T_{\min}$	99.8	91.2	89.2	88.5	88.9
	ΔT	4.7	13.1	15.3	15.9	15.7
End section of BIMC	$T_{\max}$	104.1	103.8	104.2	104.3	104.2
	$T_{\min}$	102.3	100.7	93	94.3	93.4
	ΔT	1.8	3.1	11.2	10	10.8
Intermediate section of STMC	$T_{\max}$	104.1	104.5	104	104.8	104.5
	$T_{\min}$	102.3	90.5	90.8	89.8	90.1
	ΔT	1.8	14	13.2	15	14.4
End section of STMC	$T_{\max}$	104.5	104.7	105	102.2	101.6
	$T_{\min}$	97.2	92.5	90.8	90.1	90.1
	ΔT	7.3	12.2	14.2	12.1	11.5
